# Supplementary material for: The impact of unemployment benefits on birth outcomes: Quasi-experimental evidence from European linked register data
Source: PLoS One. 2022 Mar 2;17(3):e0264544. doi: 10.1371/journal.pone.0264544 (PMC8890730; doi:10.1371/journal.pone.0264544)
Supplement: S1 Table — (DOCX) [file pone.0264544.s001.docx]

**Table S1: Regression coefficients underlying Fig 1 (left-hand panel), estimates for children with an unemployed mother.**

|  | Birth weight (g) DiD | Birth weight (g) DiD, adjusted | Body length (cm) DiD | Body length (cm) DiD, adjusted | Birth weight (g) difference to preceding sibling DiD | Birth weight (g) difference to preceding sibling DiD, adjusted | Body length (cm) difference to preceding sibling DiD | Body length (cm) difference to preceding sibling DiD, adjusted |
| --- | --- | --- | --- | --- | --- | --- | --- | --- |
| Intercept | 3299.665 *** | 2297.274 *** | 49.187 *** | 44.794 *** | 45.100 * | 137.599 | 0.096 | 0.030 |
|  | (12.159) | (72.896) | (0.062) | (0.377) | (17.726) | (92.745) | (0.098) | (0.516) |
| Treated (vs. controls) | 22.285 | 25.533 | 0.264 ** | 0.278 ** | 76.621 * | 80.402 * | 0.345 + | 0.378 * |
|  | (18.534) | (17.962) | (0.095) | (0.093) | (32.830) | (33.296) | (0.182) | (0.185) |
| Post (vs. pre) | -8.313 | 19.716 | 0.149 + | 0.266 *** | 44.682 * | 41.358 * | 0.183 | 0.187 |
|  | (15.723) | (15.079) | (0.080) | (0.078) | (20.350) | (20.538) | (0.113) | (0.114) |
| DiD (treatedXpost) | -45.647 + | -48.193 * | -0.467 *** | -0.462 *** | -116.540 ** | -110.761 ** | -0.685 ** | -0.682 ** |
|  | (25.580) | (24.248) | (0.131) | (0.125) | (40.501) | (40.570) | (0.225) | (0.226) |
| Income bef. unempl. |  | -0.003 |  | -0.000 |  | 0.001 |  | 0.000 |
|  |  | (0.004) |  | (0.000) |  | (0.005) |  | (0.000) |
| Working hours bef. unempl. |  | -0.083 |  | 0.001 |  | 0.331 |  | 0.002 |
|  |  | (0.311) |  | (0.002) |  | (0.409) |  | (0.002) |
| Hh income bef. unempl. |  | 0.002 |  | 0.000 |  | -0.001 |  | -0.000 |
|  |  | (0.001) |  | (0.000) |  | (0.002) |  | (0.000) |
| Married (vs. unmarried) |  | 92.171 *** |  | 0.350 *** |  | -23.507 |  | 0.040 |
|  |  | (15.491) |  | (0.080) |  | (24.339) |  | (0.135) |
| Managerial occupation (vs. intermediate) |  | 24.946 |  | 0.257 ** |  | -19.903 |  | 0.015 |
|  |  | (19.093) |  | (0.099) |  | (24.897) |  | (0.138) |
| Manual occupation (vs. intermediate) |  | -16.647 |  | -0.097 |  | -16.931 |  | -0.161 |
|  |  | (15.500) |  | (0.080) |  | (21.786) |  | (0.121) |
| Tertiary education (vs. vocational) |  | 26.624 |  | 0.189 * |  | 8.113 |  | 0.041 |
|  |  | (18.012) |  | (0.093) |  | (23.729) |  | (0.132) |
| Compulsory education (vs. vocational) |  | -53.479 *** |  | -0.252 ** |  | -17.423 |  | 0.044 |
|  |  | (15.293) |  | (0.079) |  | (21.679) |  | (0.121) |
| Age mother (years) |  | -6.069 *** |  | -0.037 *** |  | 3.736 |  | -0.005 |
|  |  | (1.703) |  | (0.009) |  | (2.459) |  | (0.014) |
| Age father (years) |  | -0.182 |  | 0.010 + |  | -2.878 + |  | 0.002 |
|  |  | (1.094) |  | (0.006) |  | (1.740) |  | (0.010) |
| Mother swiss (vs. non-swiss) |  | -28.748 * |  | -0.080 |  | 10.271 |  | 0.072 |
|  |  | (13.496) |  | (0.070) |  | (17.859) |  | (0.099) |
| Father swiss (vs. non-swiss) |  | -22.131 + |  | -0.004 |  | -11.996 |  | -0.073 |
|  |  | (13.177) |  | (0.068) |  | (17.421) |  | (0.097) |
| Singleton birth (vs. multiple) |  | 1041.644 *** |  | 4.630 *** |  |  |  |  |
|  |  | (35.433) |  | (0.183) |  |  |  |  |
| Parity |  | 65.076 *** |  | 0.205 *** |  | -45.474 ** |  | -0.049 |
|  |  | (7.769) |  | (0.040) |  | (14.247) |  | (0.079) |
| Mittelland (vs. Leman) |  | -24.587 |  | -0.531 *** |  | -13.115 |  | 0.110 |
|  |  | (17.420) |  | (0.090) |  | (23.802) |  | (0.132) |
| Northwest (vs. Leman) |  | 40.761 * |  | -0.027 |  | 34.835 |  | 0.283 + |
|  |  | (19.877) |  | (0.103) |  | (26.036) |  | (0.145) |
| Zurich (vs. Leman) |  | 44.158 * |  | 0.183 * |  | -30.498 |  | -0.058 |
|  |  | (17.747) |  | (0.092) |  | (23.781) |  | (0.132) |
| East (vs. Leman) |  | -8.710 |  | -0.182 + |  | -33.185 |  | 0.139 |
|  |  | (20.952) |  | (0.108) |  | (28.448) |  | (0.158) |
| Central (vs. Leman) |  | 62.002 * |  | -0.012 |  | 19.507 |  | 0.103 |
|  |  | (25.935) |  | (0.134) |  | (34.228) |  | (0.190) |
| Ticino (vs. Leman) |  | -37.745 |  | -0.082 |  | 37.941 |  | 0.108 |
|  |  | (27.569) |  | (0.143) |  | (38.707) |  | (0.215) |
| N | 8626 | 8626 | 8626 | 8626 | 4734 | 4734 | 4734 | 4734 |
| R2 | 0.001 | 0.108 | 0.002 | 0.085 | 0.002 | 0.009 | 0.002 | 0.005 |
| logLik | -66956.202 | -66468.583 | -21434.347 | -21057.755 | -36437.555 | -36420.497 | -11848.052 | -11841.952 |
| AIC | 133922.404 | 132987.166 | 42878.695 | 42165.509 | 72885.109 | 72888.995 | 23706.104 | 23731.905 |
| *** p < 0.001; ** p < 0.01; * p < 0.05; + p < 0.1. | | | | | | | | |
| Sample: Children whose first month of gestation was between month 9 and 23 after unemployment start. Unemployed mothers with at least 9 months of unemployment. Treated: 12 to 17 months with UI contributions. Controls: 18 to 23 months of UI contributions. Pre unemployment start July 2003-March 2009. Post unemployment start October 2010-August 2016. Standard errors in parentheses. | | | | | | | | |
