# Supplementary material for: The impact of unemployment benefits on birth outcomes: Quasi-experimental evidence from European linked register data
Source: PLoS One. 2022 Mar 2;17(3):e0264544. doi: 10.1371/journal.pone.0264544 (PMC8890730; doi:10.1371/journal.pone.0264544)
Supplement: S2 Table — 8 months unemployment duration instead of 9. (DOCX) [file pone.0264544.s002.docx]

**Table S2: Birth outcomes of children of unemployed mothers, controls and treated, pre and post-reform means, difference-in-differences estimates, and 95% confidence intervals of the effect of the reform. 8 months unemployment duration instead of 9.**

|  | **Controls, pre** | **Controls, post** | **Treated, pre** | **Treated, post** | **DiD estimate** |
| --- | --- | --- | --- | --- | --- |
| **Level** |  |  |  |  |  |
| Birth weight (g) | 3301.1 | 3292.5 | 3325.2 | 3263.7 | -48.2 * (-91.7;-4.8) |
| Body length (cm) | 49.2 | 49.3 | 49.5 | 49.1 | -0.4 *** (-0.7;-0.2) |
| N | 2568 | 3761 | 1923 | 1823 | 10075 |
|  |  |  |  |  |  |
| **Difference to preceding sibling** |  |  |  |  |  |
| Birth weight (g) | 45.6 | 84.8 | 126.3 | 57.5 | -104.4 ** (-180;-28.7) |
| Body length (cm) | 0.1 | 0.3 | 0.5 | 0 | -0.7 ** (-1.1;-0.3) |
| N | 1012 | 3218 | 401 | 703 | 5334 |
|  |  |  |  |  |  |
| Sample: Children whose first month of gestation was between month 8 and 23 after unemployment start. Parents with at least 8 months of unemployment. Treated: 12 to 17 months with UI contributions. Controls: 18 to 23 months of UI contributions. Pre unemployment start July 2003-March 2009. Post unemployment start October 2010-August 2016. DiD estimates are adjusted for control variables listed in Table 1. P-value thresholds DID: + = 10%, * = 5 %, ** = 1 %, *** = 0,1 %. | | | | | |
