# Supplementary material for: The impact of unemployment benefits on birth outcomes: Quasi-experimental evidence from European linked register data
Source: PLoS One. 2022 Mar 2;17(3):e0264544. doi: 10.1371/journal.pone.0264544 (PMC8890730; doi:10.1371/journal.pone.0264544)
Supplement: S3 Table — 10 months unemployment duration instead of 9. (DOCX) [file pone.0264544.s003.docx]

**Table S3: Birth outcomes of children of unemployed mothers, controls and treated, pre and post-reform means, difference-in-differences estimates, and 95% confidence intervals of the effect of the reform. 10 months unemployment duration instead of 9.**

|  | **Controls, pre** | **Controls, post** | **Treated, pre** | **Treated, post** | **DiD estimate** |
| --- | --- | --- | --- | --- | --- |
| **Level** |  |  |  |  |  |
| Birth weight (g) | 3297.9 | 3295.5 | 3319.9 | 3271.5 | -48.1 + (-99.9;3.7) |
| Body length (cm) | 49.2 | 49.4 | 49.5 | 49.2 | -0.5 *** (-0.8;-0.2) |
| N | 1846 | 2779 | 1437 | 1281 | 7343 |
|  |  |  |  |  |  |
| **Difference to preceding sibling** |  |  |  |  |  |
| Birth weight (g) | 62.2 | 94.3 | 120.8 | 53.9 | -95.3 * (-179.7;-10.9) |
| Body length (cm) | 0.2 | 0.3 | 0.4 | 0 | -0.5 * (-1;-0.1) |
| N | 804 | 2472 | 333 | 526 | 4135 |
|  |  |  |  |  |  |
| Sample: Children whose first month of gestation was between month 10 and 23 after unemployment start. Parents with at least 10 months of unemployment. Treated: 12 to 17 months with UI contributions. Controls: 18 to 23 months of UI contributions. Pre unemployment start July 2003-March 2009. Post unemployment start October 2010-August 2016. DiD estimates are adjusted for control variables listed in Table 1. P-value thresholds DID: + = 10%, * = 5 %, ** = 1 %, *** = 0,1 %. | | | | | |
