# Supplementary material for: The impact of unemployment benefits on birth outcomes: Quasi-experimental evidence from European linked register data
Source: PLoS One. 2022 Mar 2;17(3):e0264544. doi: 10.1371/journal.pone.0264544 (PMC8890730; doi:10.1371/journal.pone.0264544)
Supplement: Attachment — Submitted filename: PONE-D-21-3636 Comments.docx [file pone.0264544.s004.docx]

**PONE-D-21-36369**

I have reviewed the above submission and I have recommended acceptance with minor corrections.

1. The current results have not been discussed in detail with respect to literature.
2. Again, authors should consider discussing the results to highlight consistencies and inconsistencies of their findings.
