# Supplementary material for: The impact of unemployment benefits on birth outcomes: Quasi-experimental evidence from European linked register data
Source: PLoS One. 2022 Mar 2;17(3):e0264544. doi: 10.1371/journal.pone.0264544 (PMC8890730; doi:10.1371/journal.pone.0264544)
Supplement: Attachment — Submitted filename: Response to Reviewers.docx [file pone.0264544.s005.docx]

**Comment by editor Petri Böckerman**

There is a large literature on the complex relationship between unemployment and health (see <https://doi.org/10.1002/hec.1361>).

*As a main goal of unemployment insurance is the reduction of the adverse consequences of unemployment on well-being, we fully agree that the effects of unemployment on health is a natural starting point of our paper. In the introduction (p. 3), we therefore referenced papers on the effects of unemployment on mental health, physical health, and newborn health. We also referred to the relevance of our results for this literature in the conclusion section (p. 18).*

**Comments by Reviewer 1**

Reviewer #1: The paper is very interesting! The chosen topic is very actual! The Authors

tried to analyse a relationship between the unemployment and the social benefits.

I suggest making some small modifications:

- Line 51. Page 3. The authors wrote: "The current literature focuses on the US context and

on social programs targeting the very poorest." Please add the citations of the current

literature.

*We added a citation referencing the relevant studies.*

- What do you think about the birth outcomes? What kind of factors can influence the birth

outcomes, only the unemployment insurance? What about the mother general's health or

age? Please add some new paragraphs!

*Thank you! The paper was indeed lacking basic background information on the determinants of birth outcomes and possible mechanisms that link unemployment and unemployment insurance to birth outcomes. After the introduction (p. 5), we added a paragraph with an overview of the determinants of birthweight and empirical evidence of those determinants. We also added a paragraph on the mechanisms that link unemployment and fetal development which helps the reader to follow our paragraph on the effect of the UI reform on birth outcomes.*

**Comments by Reviewer 2**

Reviewer #2: It appears to be a sound paper. However, the current results have not been

discussed in detail with respect to literature. Again, the authors should consider discussing

the results to highlight consistencies and inconsistencies of their findings.

*We agree that in the prior version we did not pay enough attention to the implications of our results with respect to the literature. In the revision we first clarify the mechanisms underlying our results (lines 41-43; 101-119; 356-364), and second, link the paper to the literature on the determinants of birth outcomes (specifically the role of stress) and to the literature on economic shocks (specifically unemployment), stress and health (lines 90-91). In the conclusion section, we interpreted our results considering this literature, highlighting its contributions and limits to generalizability (366-383).*
